# Novel pyridine-based chalcone analogs and triple negative breast cancer: potential therapy & molecular pathways

**DOI:** 10.1038/s41598-026-51789-0

**Published:** 2026-05-30

**Authors:** Arij Fouzat Hassan, Ola J. Hussein, Hadeel Kheraldine, Sumayyah Saeed, Maysaloun Merhi, Jericha Miles Mateo, Hamda Al-Thawadi, Feras Alali, Ala-Eddin Al-Moustafa, Ashraf Khalil, Abdelbary Elhissi

**Affiliations:** 1 College of Pharmacy, Pharmaceutical Sciences Department, QU Health, Qatar University, Doha, Qatar; 2 College of Medicine, QU Health, Qatar University, Doha, Qatar; 3 National Center for Cancer Care and Research, Hamad Medical Corporation, Doha, Qatar; 4 Translational Cancer Research Facility, Interim Translational Research Institute, Hamad Medical Corporation, Doha, Qatar; 5 Oncology Department, McGill University, Montreal, QC Canada; 6 ABS Research Review & Consultation, Montreal, QC Canada; 7 College of Pharmacy, Dubai Medical University, Dubai, 19099 United Arab Emirates; 8 Biomedical Research Center, QU Health, Qatar University, Doha, Qatar

**Keywords:** Triple negative breast cancer (TNBC), Chalcones, Apoptosis, EGFR, ERK1/2, Biochemistry, Cancer, Chemical biology, Chemistry, Drug discovery

## Abstract

Triple-negative breast cancer (TNBC) represents one of its most aggressive subtypes, characterized by poor prognosis and resistance to conventional therapies. Given the urgent need for more effective treatment strategies, chalcone derivatives have attracted attention as versatile scaffolds with multitarget anticancer potential. In this study, we evaluated the anticancer efficacy of 14 novel pyridine-based chalcone analogs against TNBC cell lines (MDA-MB-231 and BT-20). Following initial screening, **OH17** and **OH25** showed promising activity and were selected for further evaluation alongside docetaxel (DTX), a standard chemotherapeutic control. Both OH17 and OH25 significantly reduced TNBC cell viability at low micromolar concentrations. Morphological analysis revealed apoptosis-like features, further supported by cell cycle analysis and Annexin V/PI staining, which demonstrated sub-G1 accumulation and induction of apoptosis. Molecular pathway analysis showed that the compounds upregulated the pro-apoptotic protein BAX while downregulating ERK1/2, with **OH17** showing additional EGFR modulation. These effects translated into significant inhibition of cell invasion and colony formation, underscoring their potential to suppress key hallmarks of TNBC aggressiveness. Collectively, our findings highlight **OH17** and **OH25** as promising chalcone analogs with potent and mechanistically distinct therapeutic activity against TNBC. These results support further preclinical evaluation of pyridine chalcones as next-generation candidates for TNBC therapy.

**Supplementary Information:**

The online version contains supplementary material available at 10.1038/s41598-026-51789-0.

## Introduction

Breast cancer continues to represent a major global health burden. Its intricate tumorigenesis and diverse clinical phenomena make it quite challenging for both treatment and prevention^1^. In 2022, breast cancer accounted for approximately 2.3 million new cases and 666,000 deaths worldwide, representing 23.8% of all cancer diagnoses and 15.4% of cancer-related deaths among women^2^. Globally, the mortality-to-incidence ratio, which is a key indicator of 5-year survival, was estimated at 0.30 in 2020, reflecting persistent disparities in outcomes across regions^3^. With the growing prevalence of breast cancer around the world, more insights into the complex nature of this disease are required to develop effective therapeutic strategies^4^. Receptors, including estrogen (ER), progesterone (PR), and human epidermal growth factor receptor 2 (HER2), are the basis for tumor subtyping, and their consideration is critical in guiding treatment decisions^5^. However, triple-negative breast cancer (TNBC) is considered the most aggressive subtype of breast cancer due to the absence of the ER, PR, and HER2 receptors^6,7^. TNBC accounts for more than 15% of all breast cancers and has a worse prognosis with higher rates of recurrence and mortality^8^. In late stages, median overall survival is just 12–18 months. Unlike other subtypes of breast cancer, TNBC tends to be diagnosed at a younger age and most commonly exhibits high-grade invasive ductal carcinoma^9^. Additionally, TNBC is highly invasive, with approximately 46% of patients developing distant metastases^10^. It is primarily treated with systemic chemotherapy due to the lack of actionable molecular targets^11^.

Current standard regimens include anthracycline and cyclophosphamide-based combinations, typically followed by taxanes such as paclitaxel or docetaxel. In addition, taxane–platinum combinations, including carboplatin- or cisplatin-containing protocols, are also widely used^12,13^. Despite these therapeutic advances, significant limitations persist, including cumulative toxicity such as cardiotoxicity and myelosuppression associated with anthracyclines, as well as increased hematological adverse effects with platinum agents^14^. Besides, the frequent development of drug resistance, particularly to taxanes, further compromises treatment efficacy^15^. Moreover, high relapse rates and variable long-term survival outcomes remain major challenges in TNBC management^16^. Taken together, these limitations underscore the urgent need for the development of novel, safer, and more effective therapeutic agents.

Chalcones, also known as 1,3-diphenyl-2-propen-1-ones, are an attractive class of open-chain flavonoids that are being heavily investigated as potential therapeutic agents for cancer^17,18^. These compounds have long fascinated medicinal chemists due to their relatively straightforward synthesis, wide range of modification possibilities, limited DNA interaction, and low mutagenic risk^19,20^. Chalcones are considered phytochemicals that possess a diverse spectrum of biological activities, including antioxidant^21^, antimicrobial^22^, antiviral^23^, anti-inflammatory^24^, antidiabetic^25^, and anticancer^26^. Notably, chalcones have demonstrated anticancer effects against TNBC, influencing key hallmarks such as proliferation, apoptosis, cell cycle, autophagy, angiogenesis, epithelial–mesenchymal transition (EMT), and metastasis^27^. They have emerged as versatile anticancer scaffolds due to their ability to modulate multiple molecular targets and signaling pathways implicated in tumor progression^26,28^. Studies have shown that chalcone derivatives can inhibit key pathways such as PI3K/AKT/mTOR, NF-κB, and MAPK^29^, while also inducing apoptosis through activation of caspases and disruption of mitochondrial membrane potential^30,31^. In addition, chalcones have been reported to target tubulin polymerization, suppress angiogenesis via VEGFR-2 inhibition^32^, and modulate cell cycle regulators including cyclins and CDKs^33^. Moreover, chalcone derivatives have been shown to inhibit EGFR signaling and its downstream MAPK/ERK cascade, while also suppressing c-Jun activation, collectively contributing to reduced proliferation and enhanced apoptosis in cancer cells^34,35^. These multitarget mechanisms are particularly relevant in TNBC, which is characterized by pathway heterogeneity and the absence of defined therapeutic targets.

Recently, we developed and patented a library of pyridine-based chalcone derivatives that showed potential anticancer activity against prostate cancer models^36^. As such, we aim in this study to assess the effectiveness of these chalcone compounds against TNBC in-vitro using MDA-MB-231 and BT-20 cell lines and to identify their molecular targets.

## Results

**Fig. 1:**
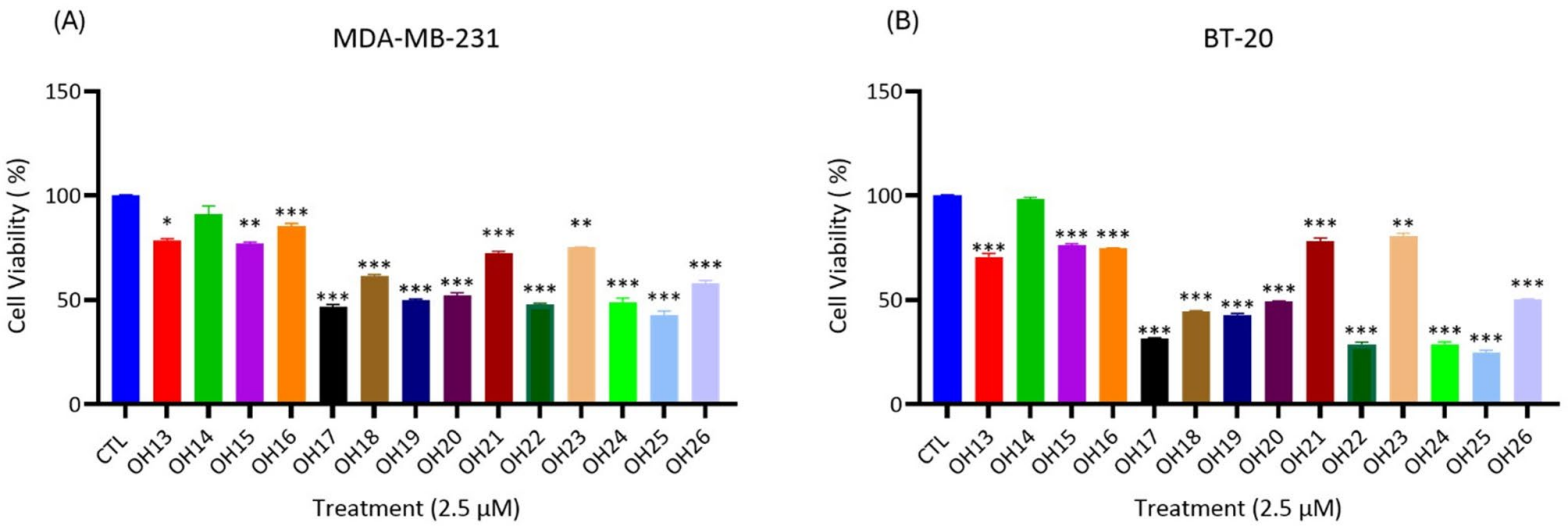
The effect of thienyl-pyridine-based chalcone derivatives (**OH13** -**OH26**) on the cell viability of (**A**) MDA-MB-231 and (**B)** BT-20 at 2.5 µM. Cell viability was determined relative to control (CTL) after 48 h of treatment. One-way ANOVA followed by Tukey’s post-hoc test was used to compare the treatment groups with untreated control (CTL). Data are presented as mean ± SEM (*n* = 3). Results were considered statistically significant when *p* < 0.05. *p < 0.05, **p < 0.01 and ***p < 0.001.

In this study, fourteen thienyl-pyridine-based chalcone derivatives (**OH13**-**OH26**) were initially screened for anticancer activity against two TNBC cell lines, MDA-MB-231 and BT-20, at a fixed concentration of 2.5 µM for 48 h. As anticipated, treatment with these compounds significantly reduced the viability of both TNBC cells in a dose-dependent manner (Fig. 1). Following initial screening, **OH17** and **OH25** were prioritized for further evaluation as representative compounds showing promising activity in TNBC cell line. In MDA-MB-231 cells, **OH17** and **OH25** significantly reduced cell viability to 46.5% and 42.8%, respectively (Fig. 1A), whereas in BT-20 cells, cell viability was reduced to 31.41% and 24.9%, respectively at 2.5 µM (Fig. 1B).

**Fig. 2:**
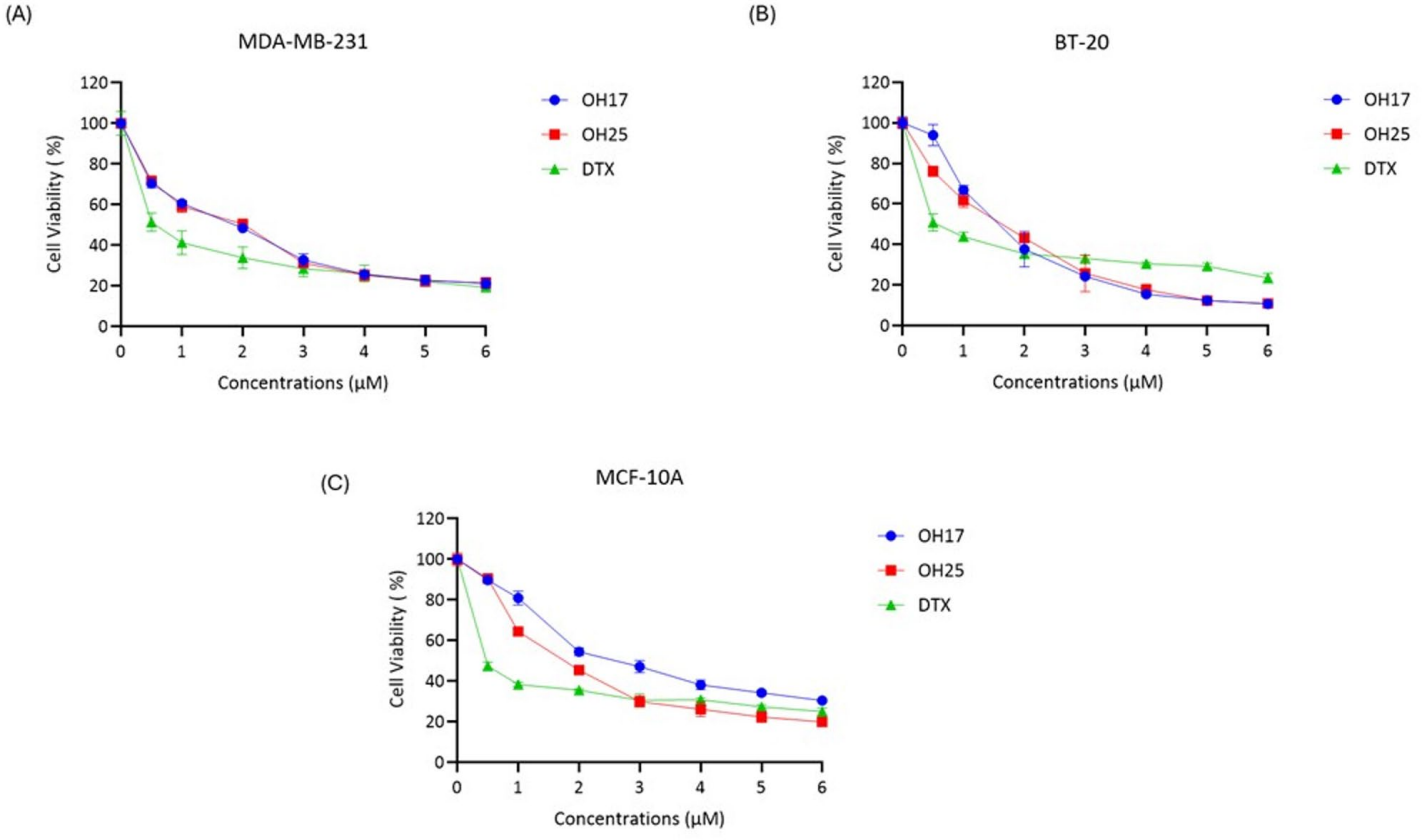
Dose-dependent cytotoxicity of **OH17**, **OH25** and DTX in TNBC cells (**A**) MDA-MB-231, (**B**) BT-20 and non-cancerous cells (**C**) MCF-10 A. All cell lines were treated with increasing concentrations (0–6 µM) of **OH17**, **OH25**, and DTX for 48 h. Cell viability was expressed as a percentage relative to untreated control (CTL). One-way ANOVA followed by Tukey’s post-hoc test was used to compare the treatment groups with untreated control (CTL). Data are presented as mean ± SEM (*n* = 3). Results were considered statistically significant when *p* < 0.05. *p < 0.05, **p < 0.01 and ***p < 0.001.

To further validate the contribution of **OH17** and **OH25** to anticancer efficacy, we compared their effects quantitatively by calculating the half-maximal inhibitory concentration (IC₅₀) of both compounds against TNBC cells. As a reference, docetaxel (DTX), a standard chemotherapeutic agent and first-line therapy for TNBC, was included as a positive control using same concentrations^37^. As shown in Fig. 2, both **OH17** and **OH25** inhibited TNBC cell viability in a concentration-dependent manner, with IC₅₀ values ranging between approximately 1.6 µM and 2 µM in MDA-MB-231 and BT-20 cells (Table 1).

**Table 1:** The IC₅₀ values of **OH17**, **OH25** and DTX in TNBC cell lines. Data are presented as mean ± SEM (*n* = 3).

Cell line	IC_50_ OH17	IC_50_ OH25	IC_50_ DTX
MDA-MB-231	2.1µM ± 1.8	2.1µM ± 1.8	0.5µM ± 0.6
BT-20	1.6µM ± 0.2	1.5µM ± 0.3	0.6µM ± 0.2

In parallel, to assess selectivity toward malignant cells, the cytotoxic effects of **OH17** and **OH25** were evaluated in the non-tumorigenic human breast epithelial cell line MCF-10 A, using the same concentration range. As shown in Fig. 2C, both compounds exhibited a dose-dependent reduction in MCF-10 A cell viability; however, they were less cytotoxic to MCF-10 A than DTX across most tested concentrations. The IC_50_ values in MCF-10 A were determined to be approximately 3 µM ± 0.1 for **OH17**, 2 µM ± 0.08 for **OH25**, and notably lower at 0.2 ± 0.07µM for DTX. Additionally, the calculated selectivity indices indicated that **OH17** and **OH25** were generally less toxic than DTX (Table 2). Based on the IC₅₀ values of **OH17** and **OH25**, a concentration of 2 µM was selected for all subsequent experiments across all compounds, including DTX.

**Table 2:** SI values of **OH17**, **OH25**, and DTX in TNBC cell lines relative to MCF-10 A.

Cell line	SI OH17	SI OH25	SI DTX
MCF-10 A/MDA-MB-231	1.5 ± 0.2	1 ± 0.06	0.4 ± 0.1
MCF-10 A/BT-20	2 ± 0.3	1.3 ± 0.09	0.3 ± 0.08

**Fig. 3:**
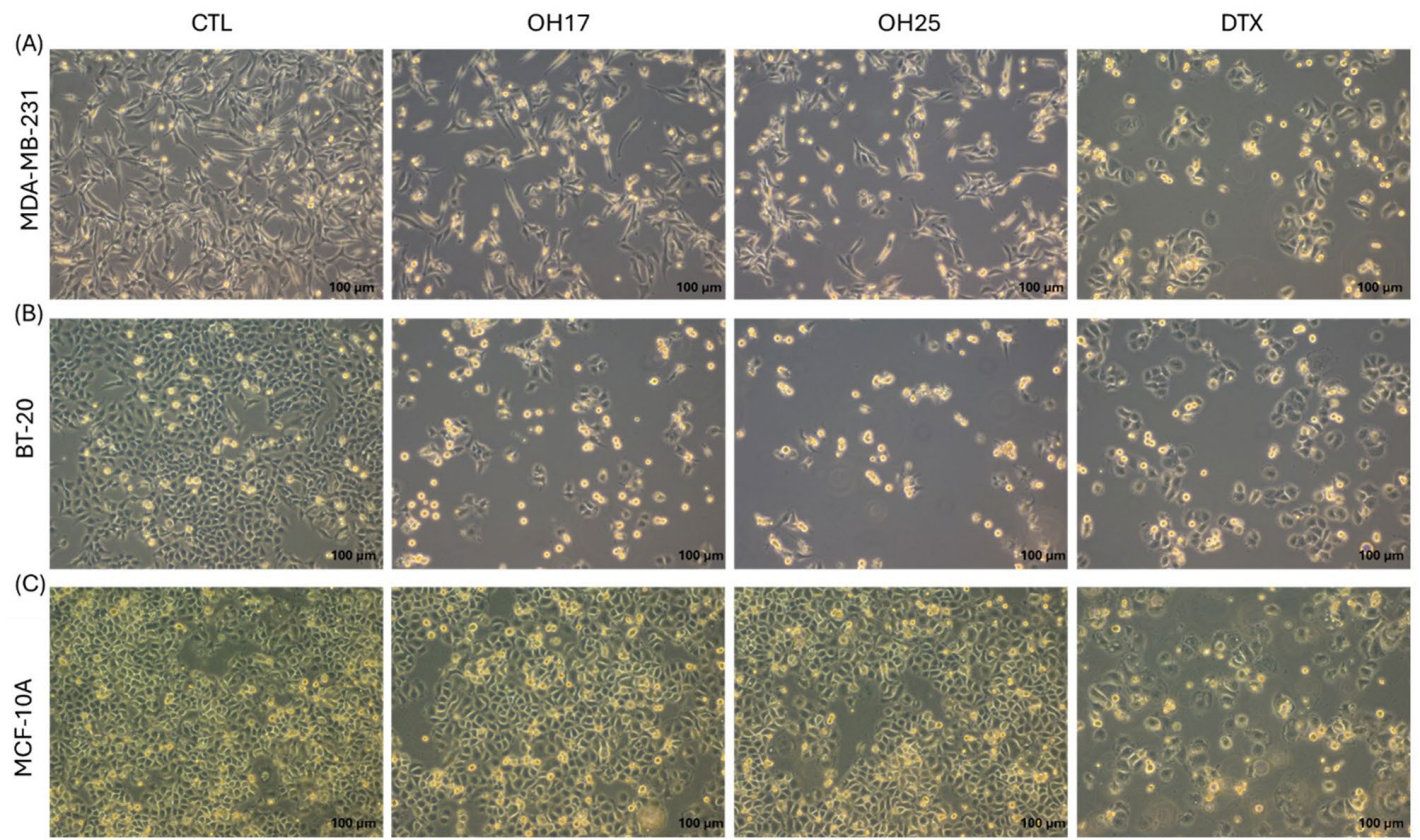
Effects of **OH17**, **OH25** and DTX (**A**) MDA-MB-231 and (**B**) BT-20 and (**C**) MCF-10 A cell lines morphological features. Cells were treated with 2 µM of **OH17**, **OH25** and DTX. Cells were treated for 48 h and images were taken using a 10X objective (*n* = 3). The scale bar represents 100 μm.

Next, the effect of **OH17** and **OH25** at 2 µM on cellular morphology was examined following 48 h of treatment compared to untreated control and DTX (2 µM). As shown in Fig. 3A, untreated MDA-MB-231 cells displayed elongated, spindle-shaped morphology with dense monolayer formation, whereas treatment with **OH17** or **OH25** resulted in pronounced morphological alterations, including cell rounding, shrinkage, membrane blebbing, and detachment. Comparable morphological changes and reductions in cell density were observed in BT-20 cells following treatment, where untreated BT-20 cells maintained their typical polygonal, cobblestone-like epithelial morphology (Fig. 3B). In contrast, MCF-10 A cells retained normal morphology after exposure to **OH17** or **OH25**, with only minor reductions in cell density and occasional rounding (Fig. 3C). DTX treatment induced marked morphological disruption in TNBC and MCF-10 A cells.

**Fig. 4:**
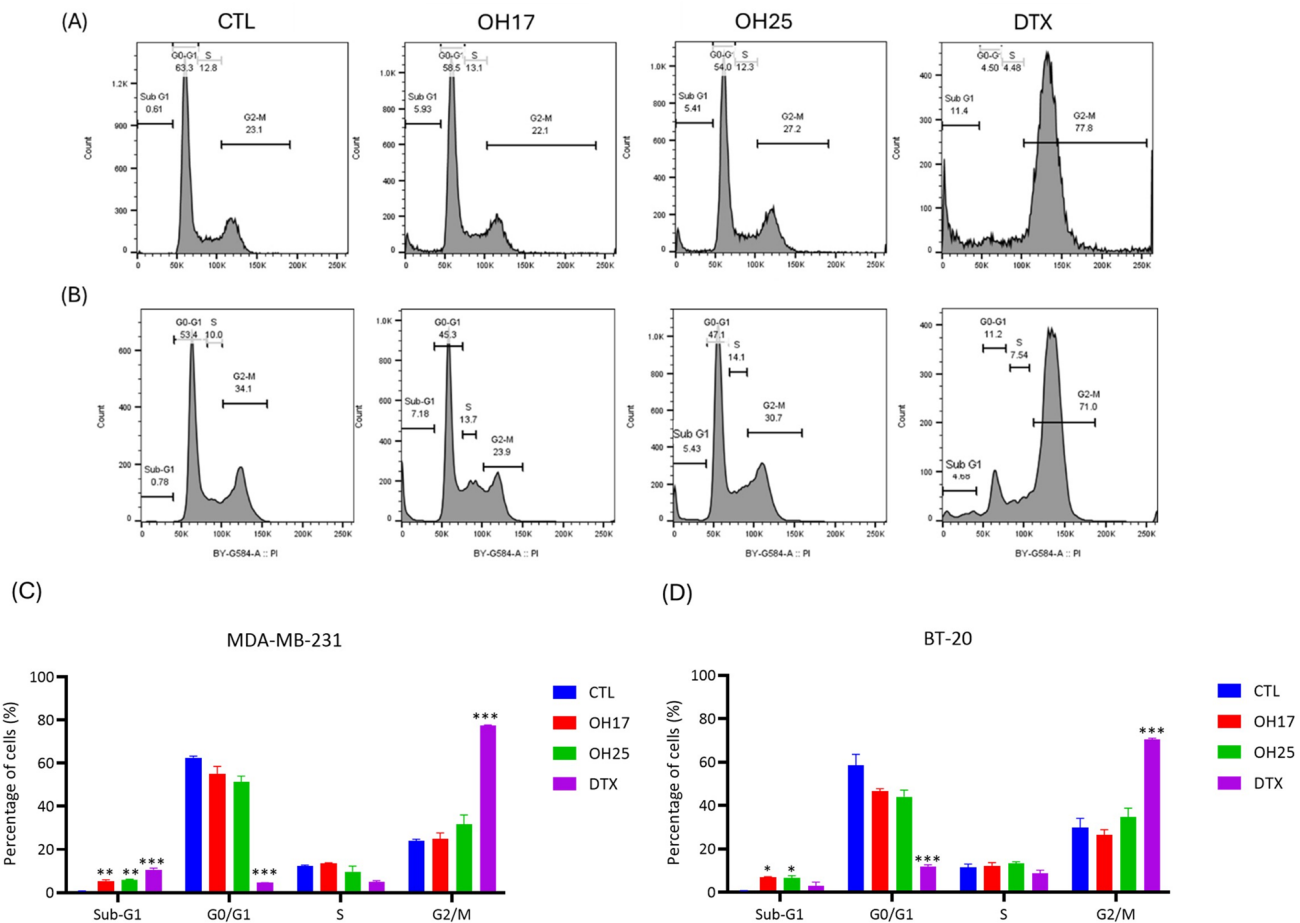
Effect of **OH17**, **OH25** and DTX on cell-cycle phase distribution in (**A**, **C**) MDA-MB-231 and (**B**, **D**) BT-20 cell lines at 2µM concentration for 48 h. Flowcytometry analysis and histograms presenting the Sub-G1, G0/G1, S-phase, and G2/M cell cycle phases in cells treated with both chalcone compounds and DTX, then stained with propidium iodide (PI) as compared to untreated control (CTL). One-way ANOVA followed by Tukey’s post-hoc test was used to compare the treatment groups with untreated control (CTL). Data are presented as mean ± SEM (*n* = 3). Results were considered statistically significant when *p* < 0.05. *p < 0.05, **p < 0.01 and ***p < 0.001.

**Fig. 5:**
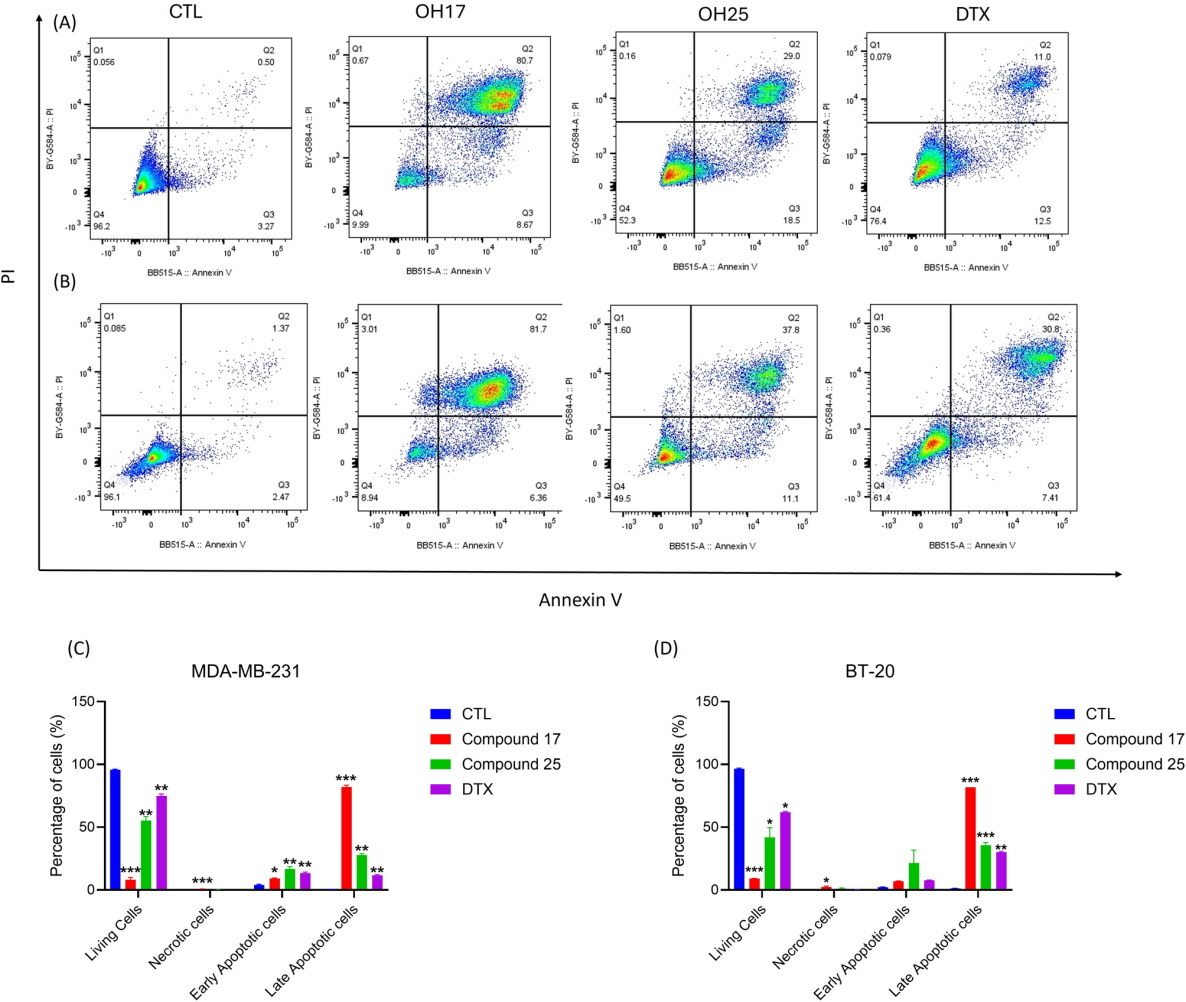
Annexin V/PI apoptosis analysis of (**A**, **C**) MDA-MB-231 and (**B**, **D**) BT-20 cells treated with **OH17**, **OH25** and DTX at 2µM concentration for 48 h. Representative flow cytometry dot plots (A) MDA-MB-231 and (B) BT-20 show viable cells (Annexin V⁻/PI⁻, lower left quadrant), early apoptotic cells (Annexin V⁺/PI⁻, lower right), late apoptotic/secondary necrotic cells (Annexin V⁺/PI⁺, upper right), and necrotic cells (Annexin V⁻/PI⁺, upper left). Quantification of apoptotic distribution is presented in the accompanying bar graphs (C) MDA-MB-231 and (D) BT-20. One-way ANOVA followed by Tukey’s post-hoc test was used to compare the treatment groups with untreated control (CTL). Data are presented as mean ± SEM (*n* = 3). Results were considered statistically significant when *p* < 0.05. *p < 0.05, **p < 0.01 and ***p< 0.001.

To further characterize the impact of these compounds on TNBC, flowcytometry analysis of cell cycle distribution and apoptosis was conducted. **OH17** and **OH25** induced a substantial increase in the sub-G1 population in both MDA-MB-231 and BT-20 cells (Fig. 4A). By comparison, DTX treatment resulted in a prominent accumulation of cells in the G2/M phase. Consistent with the observed increase in the sub-G1 population, treatment with both compounds resulted in a marked increase in Annexin V–positive cells, supporting the induction of apoptosis (Fig. 5).

**Fig. 6:**
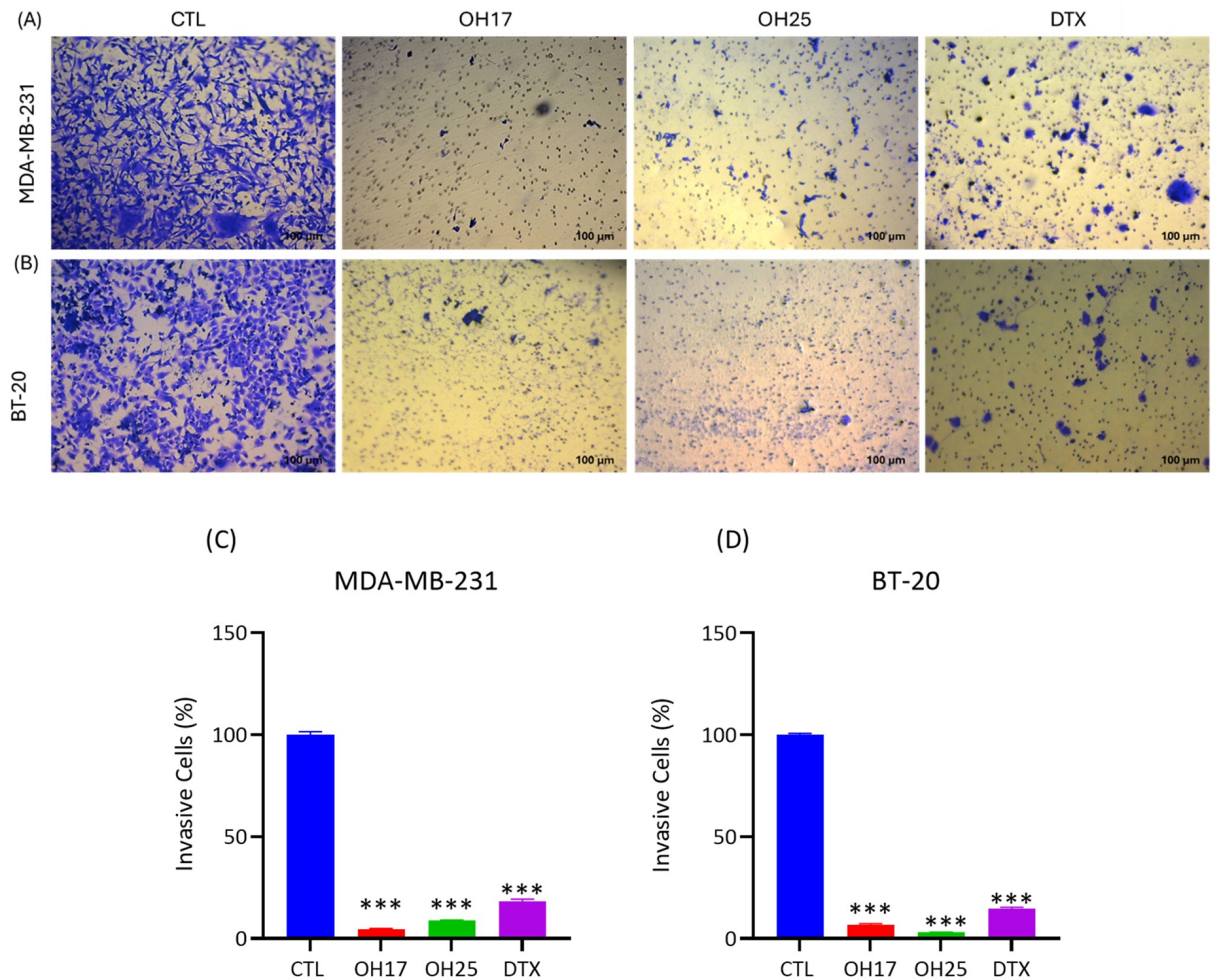
Effect of **OH17**, **OH25** and DTX on cell invasion on (**A**, **C**) MDA-MB-231 and (**B**, **D**) BT-20 cell lines. A, B) Images were taken following 48 h of treatment with 2 µM concentration of **OH17**, **OH25**, and DTX. Invaded cells were quantified compared to the untreated control (CTL) (C, D). One-way ANOVA followed by Tukey’s post-hoc test was used to compare the treatment groups with untreated control (CTL). Data are presented as mean ± SEM (*n* = 3). Results were considered statistically significant when *p* < 0.05. ***p < 0.001. Images were taken using a 10X objective (*n* = 3). The scale bar represents 100 μm.

**Fig. 7:**
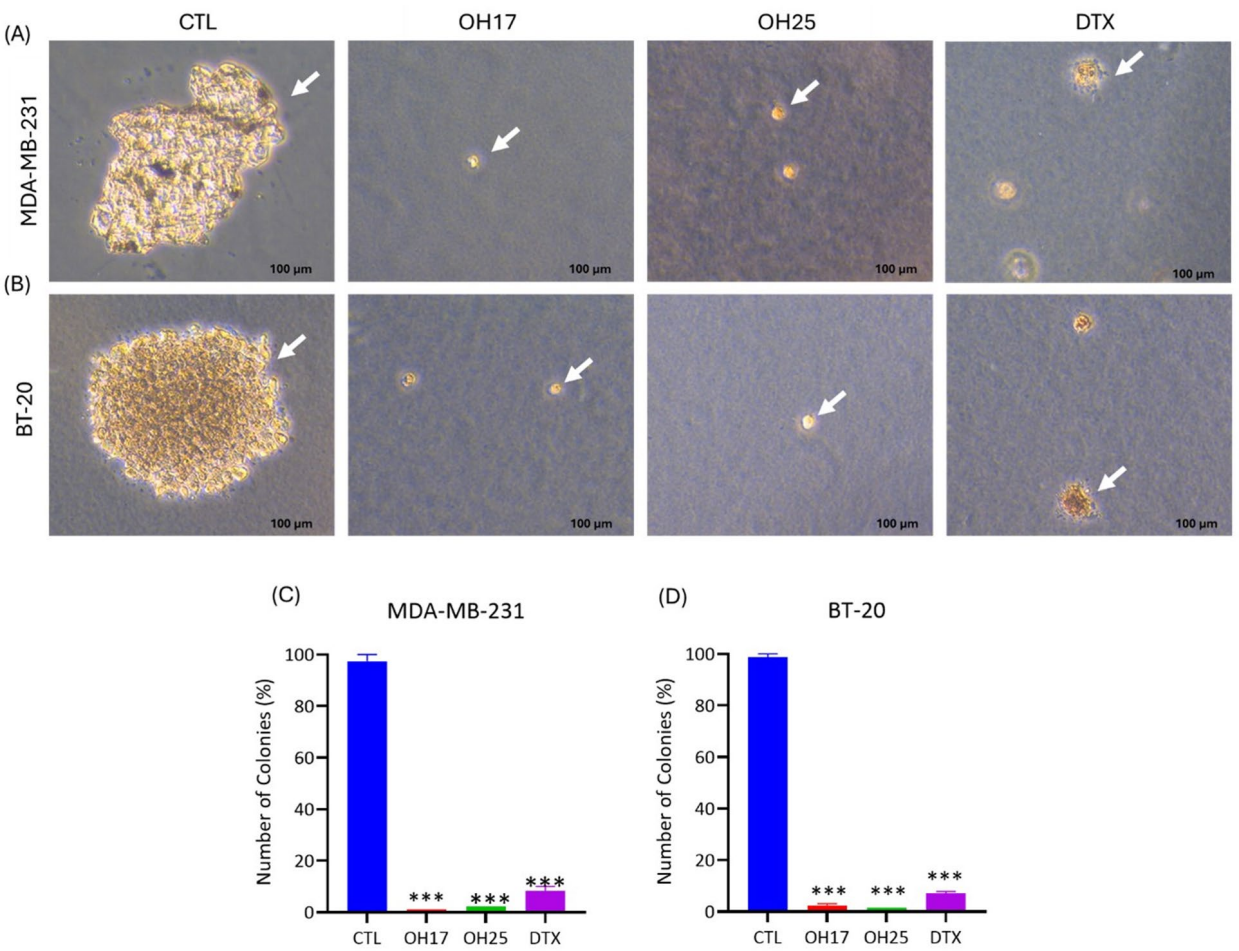
Effect of **OH17**, **OH25** and DTX at 2 µM on colony formation of (**A**, **C**) MDA-MB-231 and (**B**, **D**) BT-20 cells after 48 h. Representative images show a marked reduction in colony number and size in all treated groups compared to the control. Arrows indicate representative colonies. Colonies were counted at the end of the experiment. Representative images of colonies from each treatment group are shown. Quantification (C and D) expressed as the percentage of colonies formed relative to the untreated control (CTL) reveals a significant suppression of clonogenic capacity. One-way ANOVA followed by Tukey’s post-hoc test was used to compare the treatment groups with untreated control (CTL). Data are presented as mean ± SEM (*n* = 3). Results were considered statistically significant when *p* < 0.05. ***p < 0.001. Images were taken using a 10X objective (*n* = 3). The scale bar represents 100 μm.

Given the aggressive and metastatic nature of TNBC, the effect of **OH17** and **OH25** on cellular invasiveness was evaluated using Matrigel-coated trans well assays. As illustrated in Fig. 6, untreated MDA-MB-231 and BT-20 cells readily invaded through the matrix and were observed as dense clusters on the lower membrane surface. Treatment with **OH17** or **OH25** resulted in a marked reduction in invasion under the tested conditions. Given that these experiments were performed at a concentration (2 µM) associated with significant cytotoxic effects, this reduction may, at least in part, reflect decreased cell viability rather than a specific anti-invasive mechanism. Beyond inhibiting migratory behavior, an effective anticancer agent must also suppress the long-term proliferative potential of cancer cells. To evaluate this, we examined the effect of **OH17** and **OH25** on the ability of TNBC cells to form colonies as an indicator of their tumorigenic potential. Untreated MDA-MB-231 and BT-20 cells formed large, dense colonies, whereas treatment with both compounds resulted in a marked reduction in both colony numbers and size (Fig. 7). Quantitative analysis demonstrated greater than 95% inhibition of colony formation in both cell lines following treatment with either compound.

**Fig. 8:**
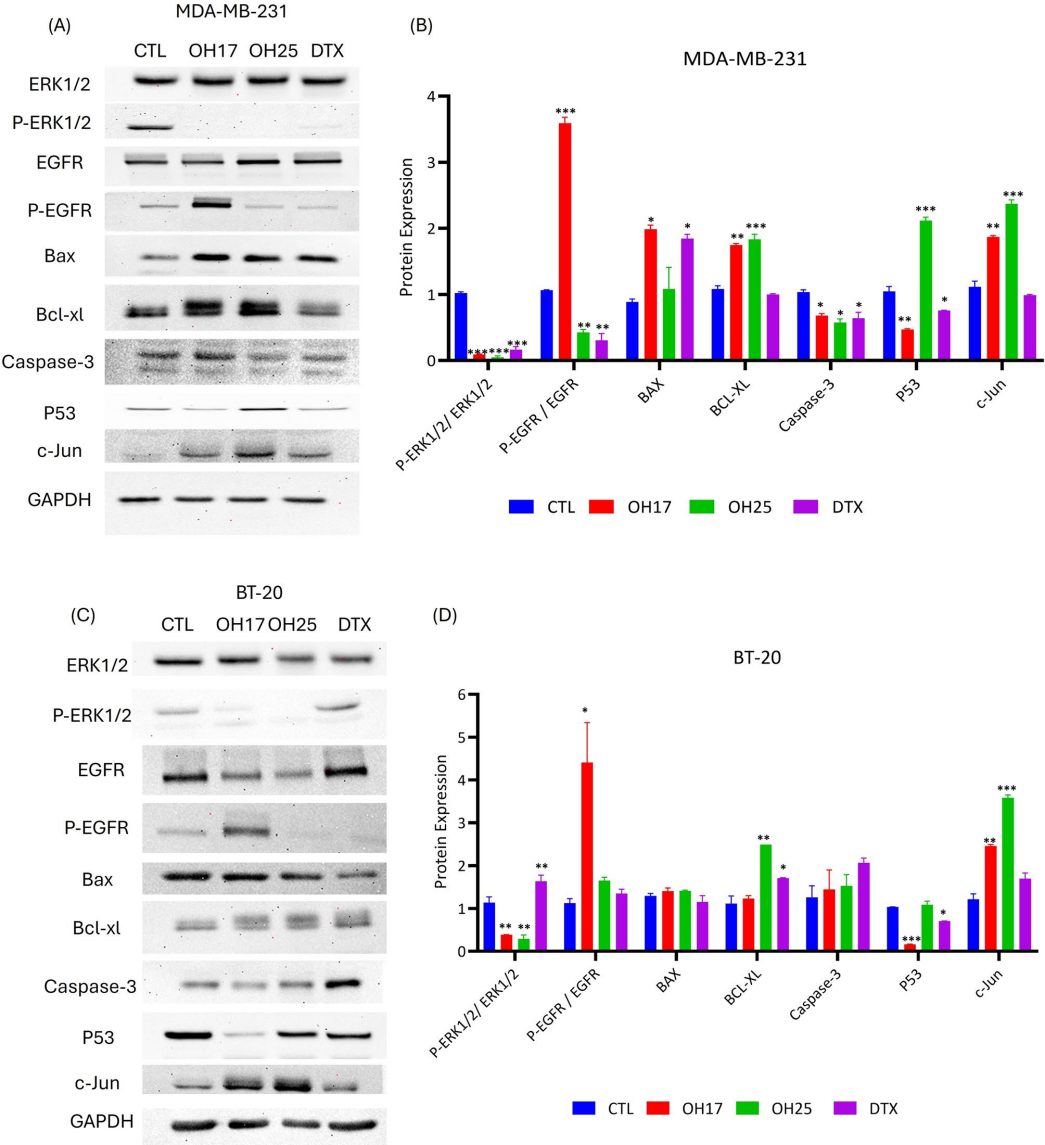
Expression of key apoptosis-related proteins in MDA-MB-231 and BT-20 cells after treatment with **OH17**, **OH25** and DTX. Cells were treated with **OH17**, **OH25** and DTX for 48 h at 2 µM concentration. GAPDH was used as a loading control. The bar graph quantifies the protein normalized to GAPDH, expressed relative to the untreated control (CTL). One-way ANOVA followed by Tukey’s post-hoc test was used to compare the treatment groups with untreated control (CTL). Data are presented as mean ± SEM (*n* = 3). Results were considered statistically significant when *p* < 0.05. *p < 0.05, **p < 0.01 and ***p < 0.001.

Later, we performed western blot analysis to examine changes in the expression of key proteins involved in the anticancer mechanisms of both chalcone compounds. Our results revealed that MDA-MB-231 and BT-20 cells have distinct and overlapping molecular responses to **OH17** and **OH25**, shedding light on their mechanisms of action. In MDA-MB-231 cells (Fig. 8A&B), treatment with both chalcone compounds suppressed ERK pathway activation, as evidenced by a significant reduction in the p-ERK1/2 to ERK1/2 ratio compared to the untreated control. In contrast, p-EGFR was significantly increased following **OH17** treatment, as indicated by a robust elevation in the p-EGFR/EGFR ratio, while **OH25** induced significant decrease. These findings suggest differential modulation of upstream EGFR signaling despite downstream ERK inhibition.

Analysis of apoptosis-related markers in MDA-MB-231 revealed that **OH17** significantly upregulated the pro-apoptotic protein Bax, whereas **OH25** exerted a nonsignificant effect. Both compounds increased the expression of the anti-apoptotic protein Bcl-xl, indicating a complex balance between pro-survival and pro-death signals. Caspase-3 expression was significantly reduced following treatment with **OH17** and **OH25**, suggesting limited activation of classical caspase-dependent apoptosis under these conditions. Notably, p53 expression was significantly elevated in response to **OH25**, but not **OH17**, while both chalcone derivatives strongly upregulated c-Jun expression, with **OH25** producing the highest induction among all treatment groups (Fig. 8A and B).

In BT-20 cells (Fig. 8C &D), similar inhibition of ERK signaling was observed. Both chalcones significantly decreased the p-ERK1/2 to ERK1/2 ratio relative to control. EGFR phosphorylation was strongly induced by **OH17**, as reflected by a marked increase in the p-EGFR to EGFR ratio, whereas **OH25** produced more modest, but nonsignificant effects. Unlike MDA-MB-231 cells, both compounds did not affect Bax expression in BT-20 cells. However, **OH25** significantly increased Bcl-xl levels, indicating enhanced activation of survival pathways. Both compounds did not produce a statistically significant effect on the expression of caspase-3. Expression of p53 was significantly downregulated following treatment with OH17, while OH25 showed no significant effect, suggesting cell-line-dependent responses. In contrast, c-Jun expression was significantly upregulated by both compounds, with **OH25** again producing the most pronounced effect.

## Discussion

In this study fourteen thienyl-pyridine-based chalcone derivatives (**OH13 **– **OH26**) were evaluated for their anticancer effect against TNBC. Although all fourteen compounds demonstrated potent activity against TNBC cell lines, compounds **OH17** and **OH25** were selected for further evaluation based on their superior efficacy. Through a sequential in-vitro evaluation, these compounds demonstrated low-micromolar cytotoxicity, and robust suppression of key aggressive cancer phenotypes, including cell cycle, apoptosis, invasion, and colony formation. Together, these findings highlight the therapeutic potential of rationally designed chalcone scaffolds as alternative agents for the treatment of aggressive TNBC.

The potent anticancer activity of **OH17** and **OH25** appear to be driven by rational structural modification of the chalcone scaffold. In addition to the thienyl-pyridine moiety, the structural basis of **OH17** and **OH25** activity may relate to the position of the methoxy substitution in **OH17** and the trifluoromethyl in **OH25**. Methoxy-substituted chalcones have frequently been reported to enhance cytotoxicity in TNBC, likely due to their electron-donating effect, which increases conjugation and facilitates interactions with cellular targets involved in proliferation and apoptosis^26,27,38^. Before the development of the current pyridine-based chalcone derivatives, our group had already designed and patented a novel nitrogen-based chalcone (DK14) bearing a methoxy substituent and a nitrogen mustard functional group. DK14 demonstrated a strong anticancer activity against TNBC both in-vitro and in-vivo, highlighting the importance of this functional group in modulating chalcones’ biological activity^32^. Consistent with these observations, previous studies have reported that incorporation of methoxy groups can markedly influence chalcone bioactivity^38–40^. A study designed poly‑methoxy chalcone compounds demonstrated that methoxy-substituted chalcones possessed potent cytotoxic effect, further supporting the notion that rational substitution on the chalcone scaffold is a key determinant of anticancer potency^41^.

Furthermore, the incorporation of trifluoromethyl substituents has also been linked to enhanced anticancer activity^42^. Several studies have further demonstrated that trifluoromethyl-bearing chalcones exhibit potent cytotoxicity against a wide range of cancer cell lines^43–46^. This may be attributed to the strong electron-withdrawing effect of the trifluoromethyl group, which can modulate the electronic distribution across the chalcone scaffold, thereby influencing binding affinity to molecular targets such as kinases and transcription factors^42,47^. Besides, pyridine-based chalcones further support this structure–activity relationship, as incorporation of a heteroaromatic pyridine ring has been shown to enhance anticancer potency by improving hydrogen-bonding capacity with proteins involved in cell survival pathways^48,49^.

Both compounds demonstrated low micromolar activity compared to those reported for other chalcones evaluated in the same cell lines. Previous reports indicate that many chalcone derivatives exhibit moderate cytotoxicity in TNBC models, with IC₅₀ values ranging from 9 µM to 50 µM for pyrrolidine-substituted derivatives in MDA-MB-231 cells, and 26.42–30.78 µM for trans-chalcone and licochalcone A in BT-20 cells^50–52^. Even the nitrogen-based chalcone previously synthesized and patented by our group, although active, displayed higher IC₅₀ values ranging between 6.3 and 9.22 µM in TNBC cells, indicating that while effective, it is still less potent than our current pyridine-based chalcones^32^.

Our morphological assessment of MDA-MB-231 and BT-20 cells following treatment with **OH17** and **OH25** revealed hallmark features consistent with apoptotic induction. These changes are in line with previous studies in TNBC cells treated with chalcones, which have documented similar features of blebbing and apoptotic bodies^32,53–56^. In contrast, these compounds caused only minimal morphological alterations in MCF-10 A cells, suggesting limited toxicity toward non-malignant epithelium cells^32,57^. However, DTX treatment caused dramatic changes in the three cell lines, consistent with DTX’s known mechanism of microtubule stabilization and induction of mitotic arrest and apoptosis^58^. Together with the IC₅₀ and SI data, the preservation of near-normal morphology in MCF-10 A cells indicates that OH17 and OH25 exhibit a more favorable safety profile than DTX.

In line with these cytotoxic and apoptotic features, **OH17** and **OH25** resulted in a marked accumulation of cells in the sub-G1 phase, a cell cycle region typically associated with apoptotic cell death and DNA fragmentation^59,60^. This observation aligns with the morphological features observed and Annexin V/PI staining analysis. The concordant increase in sub-G1 accumulation and Annexin V/PI positivity indicates that apoptosis, rather than necrosis, is the primary mode of cell death induced by **OH17** and **OH25**. Multiple studies validated that the sub-G1 peak is an indicator of apoptotic cell death in TNBC^61,62^. Nevertheless, chalcone compounds can deregulate the cell cycle at different phases in TNBC especially in G2/M and G0/G1 phases^32,63^. Regarding DTX treatment, as expected, our results are consistent with its known mechanism of action as a microtubule-stabilizing agent that causes mitotic arrest^58,64^. Our results extend earlier work on the parent chalcone DK14 and other nitrogen-based analogs, reinforcing the chalcone scaffold as a privileged structure for apoptosis induction in TNBC^27,65,66^. Notably, the enhanced sub-G1 accumulation and Annexin V positivity observed with **OH17** and **OH25** indicate that rational ring A substitution further strengthens the pro-apoptotic capacity of this scaffold.

Moreover, **OH17** and **OH25** nearly abolished invasion and colony formation, outperforming both previously reported chalcones and DTX, a finding of translational relevance given that metastasis is the primary cause of mortality in TNBC^67,68^. While the observed reduction in invasion and clonogenic potential is consistent with previously reported effects of chalcone derivatives^32,66^, the current data do not allow definitive conclusions regarding a direct anti-metastatic mechanism. Previous studies have shown that chalcones can modulate pathways involved in invasion and metastasis, including MMP expression and EMT signaling^69,70^. However, further investigations will be required to determine whether the compounds evaluated here exert similar effects at the molecular level. While DTX can limit invasion by disrupting cytoskeletal dynamics^71,72^, the more pronounced anti-invasive effects observed with chalcones consistent with reports demonstrating strong chalcone-mediated suppression of TNBC invasion in-vitro and in-vivo^32,66,73^. Additionally, the reduction in colony formation indicates that **OH17** and **OH25** may impair clonogenic recovery under the tested conditions, whereas docetaxel has been reported to produce only partial suppression^74,75^. Resistance to DTX in TNBC has been associated with several mechanisms, including increased drug efflux mediated by ATP-binding cassette (ABC) transporters, alterations in tubulin dynamics, and evasion of apoptosis through dysregulation of Bcl-2 family proteins^76–78^. These factors can contribute to reduced treatment efficacy and tumor relapses. In the present study, the observed effects of **OH17** and **OH25** on cell viability, apoptosis, and clonogenic recovery may be relevant in this context, as modulation of apoptotic pathways could potentially counteract resistance mechanisms linked to impaired cell death signaling^79^.

Moreover, these findings align with prior reports showing that diverse chalcone derivatives, including xanthohumol, licochalcone A, and pyridine-based analogs, markedly impair long-term colony survival through apoptosis induction and inhibition of pro-survival MAPK/AKT signaling^80,81^. These effects are generally attributed to chalcone-mediated activation of apoptosis, modulation of the Bax/Bcl-2 axis, and inhibition of pro-survival MAPK/AKT signaling^82,83^.

Mechanistically, our findings demonstrate that both compounds consistently suppress ERK pathway activation. They also elicit cell-line-specific modulation of upstream EGFR signaling and downstream stress and survival responses. These results highlight the complexity of chalcone-mediated signaling effects in TNBC and suggest that growth inhibition may occur through mechanisms that extend beyond classical apoptosis. A key observation of this study is the marked reduction in ERK1/2 phosphorylation following treatment with **OH17** and **OH25** in both TNBC models. Sustained activation of the MAPK/ERK pathway is a well-established driver of proliferation, migration, and therapeutic resistance in TNBC^84^. Therefore, the ability of both chalcone derivatives to inhibit ERK activation across distinct TNBC backgrounds suggests a robust anti-proliferative mechanism^85^. Interestingly, ERK inhibition occurred despite a pronounced increase in EGFR phosphorylation, particularly following **OH17** treatment. This apparent disconnect between EGFR activation and downstream ERK signaling suggests the involvement of compensatory or non-canonical signaling mechanisms^86,87^. Similar paradoxical activation of receptor tyrosine kinases in response to cytotoxic or stress-inducing agents has been reported and is often interpreted as an adaptive cellular response rather than a direct driver of proliferation^88^. In this context, EGFR phosphorylation may reflect stress-induced receptor activation or feedback signaling that fails to propagate effectively through the MAPK cascade due to downstream blockade^89^. Such uncoupling of EGFR from ERK signaling has been described as a feature of adaptive resistance signaling in aggressive cancers, including TNBC^90,91^.

The modulation of apoptosis-related proteins further supports the notion that **OH17** and **OH25** do not primarily induce classical caspase-dependent apoptosis. In MDA-MB-231 cells, **OH17** increased Bax expression, suggesting partial engagement of pro-apoptotic signaling^92^; however, this was not accompanied by caspase-3 activation. Instead, caspase-3 levels were reduced across treatments, indicating that cell death may proceed through caspase-independent mechanisms^93^. Concurrent upregulation of the anti-apoptotic protein Bcl-xl in both cell lines further supports a model in which TNBC cells activate survival pathways to counterbalance cytotoxic stress, thereby limiting executioner caspase activation^94,95^.

The differential regulation of p53 between the two TNBC models underscores the heterogeneity of TNBC signaling responses^96^. **OH25** induced p53 upregulation in MDA-MB-231 cells suggests activation of DNA damage or stress-response pathways^97^. However, p53 downregulation in BT-20 cells highlights a cell-line-dependent response that may reflect differences in p53 regulatory networks or baseline mutational status^98^. These findings emphasize that the antitumor effects of chalcone derivatives are not mediated by a uniform apoptotic program, but rather by context-dependent stress responses that converge on growth suppression.

One notable finding of this study is the consistent upregulation of c-Jun observed in both TNBC cell lines following treatment with **OH17** and **OH25**. A central component of the AP-1 transcription factor complex is involved in cancer biology. It can function as either a pro-survival or pro-death mediator depending on the cellular context and duration of activation^99–102^. In the present study, c-Jun induction occurred alongside ERK inhibition and limited caspase activation, suggesting that c-Jun may act as a stress-response mediator rather than a proliferative driver^103^. Previous studies have shown that sustained c-Jun activation under conditions of MAPK suppression can promote growth arrest, senescence, or non-apoptotic cell death, supporting this interpretation^104,105^.

Taken together, our findings suggest that the pyridine-chalcone compounds **OH17** and **OH25** may represent promising therapeutic candidates for TNBC. These results support further in-vivo investigations to evaluate their effects on tumor growth, such as in orthotopic xenograft or transgenic mouse models, as well as additional mechanistic studies to better define their targeted signaling pathways.

## Conclusions

In conclusion, this study demonstrates the anti-tumor effects of novel pyridine-based chalcone compounds **OH17** and **OH25** in TNBC cell lines, MDA-MB-231 and BT-20, while providing initial insights into the potential signaling pathways they may target. Building on our previous work on chalcone-based nitrogen compounds in breast cancer, these findings suggest that **OH17** and **OH25** may serve as promising candidates for further investigation, particularly due to their effects on ERK and EGFR signaling pathways, which are known to play important roles in TNBC biology. Additionally, both compounds were found to reduce cell viability, induce cell cycle arrest at the sub-G1 phase, and promote apoptosis in TNBC cells. They also exhibited inhibitory effects on colony formation and cell invasion. Taken together, these results support the need for further studies to better elucidate the underlying mechanisms of action of **OH17** and **OH25**. Moreover, exploring their potential activity in other human cancer types may provide additional insight into their broader therapeutic relevance. Overall, these chalcone-based compounds may contribute to the development of future therapeutic strategies for TNBC and possibly other aggressive cancers.

## Methods

### Chemistry

All chemicals and solvents were obtained from Sigma-Aldrich (USA), unless otherwise specified. The chalcone derivatives were synthesized via a Claisen–Schmidt condensation reaction, in accordance with the method reported in our granted patent US 2025/0170111 A1^36^.

### Cell culture

TNBC cell lines, MDA-MB-231 and BT-20, and the non-cancerous human mammary epithelial cell line MCF-10 A were used in this study. All cell lines were obtained from the American Type Culture Collection (ATCC, Manassas, VA, USA) and cultured under standard aseptic conditions. All cell lines were maintained in Dulbecco’s Modified Eagle Medium (DMEM; Gibco, USA) supplemented with 10% fetal bovine serum (FBS; Gibco, USA) and 1% penicillin-streptomycin (P/S; Gibco, USA). All cells were incubated at 37 °C in a humidified atmosphere with 5% CO₂. Culture media were refreshed every 2–3 days, and cells were sub-cultured upon reaching 80–90% confluence using 0.25% trypsin-EDTA (Gibco, USA). Mycoplasma contamination was routinely monitored using ScienCell™ Mycoplasma PCR Detection Kit (Cat. #8208, ScienCell Research Laboratories, USA), following the manufacturer’s protocol to ensure the authenticity and sterility of cell cultures throughout all experiments. Only mycoplasma-free cells were used in subsequent experiments.

### Cell viability

Cell viability was assessed using the alamarBlue assay, which measures metabolic activity as an indicator of cell health. MDA-MB-231, BT-20, and MCF-10 A cells were seeded in 96-well plates at a density of 5,000 cells per well in 100 µL of complete growth medium and allowed to adhere overnight at 37 °C in a 5% CO₂ incubator. The following day, cells were treated with varying concentrations (0,1,2,3,4,5,6 µM) of the chalcone compounds diluted in complete medium. Since the test compounds were dissolved in DMSO, a vehicle control group receiving the same final DMSO concentration was included, and the DMSO level did not exceed 0.1% in any experiment. After 48 h of incubation, 10 µL of Alamar Blue reagent (Thermo Fisher Scientific, USA) was added to each well, and the plates were returned to the incubator for an additional 3 h. Fluorescence was then measured at an excitation wavelength of 560 nm and emission at 590 nm using an Infinite 200 PRO Microplate Reader (Tecan, Switzerland). Cell viability was expressed as a percentage relative to untreated control wells.

### Morphological examination

Cell morphology was monitored to assess potential cytotoxic effects of the tested compounds. MDA-MB-231, BT-20, and MCF-10 A cells were seeded in 6-well plates at a density of 2 × 10⁵ cells per well and allowed to adhere overnight under standard culture conditions (37 °C, 5% CO₂). Following treatment with 2 µM of the chalcone compounds, DTX and vehicle control for 48 h, morphological changes were observed and documented using DMi8 inverted microscope (Leica, Germany). Changes in cell shape, detachment, membrane blebbing, cell shrinkage, and loss of adherence were evaluated as indicators of cytotoxicity and apoptosis-like features. Images were captured in multiple fields per well using a camera attached to the microscope. Untreated control cells were included in each experiment for comparison.

### Cell cycle

Cell cycle distribution was assessed using flow cytometry following propidium iodide (PI) staining. MDA-MB-231 and BT-20 cells were seeded in petri dishes at a concentration of 1 × 10^6^ cells/dish and allowed to adhere overnight under standard culture conditions. The cells were serum-starved to achieve cell-cycle synchronization. Then, they were treated with 2 µM of the chalcone compounds, DTX or the control vehicle for 48 h. After treatment, cells were harvested by trypsinization, washed twice with cold phosphate-buffered saline (PBS), and fixed in 70% ethanol at − 20 °C overnight. The fixed cells were then washed again with PBS and stained with BD Pharmingen™ PI/RNase Staining Buffer (BD Biosciences, USA) for 15 min at room temperature in the dark. DNA content was analyzed using BD LSRFortessa™ Cell Analyzer (BD Biosciences, USA). The proportion of cells in Sub-G1, G0/G₁, S, and G₂/M phases was quantified using FlowJo software (V10, Tree Star, USA).

### Apoptosis (Annexin V/PI)

Apoptosis was evaluated using an Annexin V apoptosis Detection Kit (BD Pharmingen, USA) according to the manufacturer’s instructions. Briefly, TNBC cells were seeded in petri dishes at a concentration of 1 × 10^6^ cells/dish and allowed to adhere overnight under standard culture conditions. On the second day, fresh media was added, and cells were treated with 2µM of the chalcone compounds, DTX or control vehicle for 48 h. Cells were harvested following 48 h of treatment, and each group of cells was stained with Annexin V–FITC and PI as per the protocol specified on the kit for 30 min at room temperature in the dark. Samples were then analyzed by BD LSRFortessa™ Cell Analyzer (BD Biosciences, USA). The percentage of cells (living cells, necrotic cells, early apoptotic cells, and late apoptotic cells) was quantified in each of the four quadrants. Data and figures were processed using FlowJo software (V10, Tree Star, USA).

### Invasion assay

The invasive potential of MDA-MB-231 and BT-20 cells was evaluated using a Matrigel-coated trans well invasion assay. Cell invasion assay was performed using Corning^®^ BioCoat^®^ Matrigel^®^ Invasion Chambers with 8.0 μm PET Membrane (Corning, USA) as per the manufacturer’s protocol. In brief, untreated or treated cells with 2 µM of chalcone compounds or DTX were seeded in the upper insert (5 × 10^4^ cells) and maintained in DMEM serum-free medium. Complete DMEM medium with 10% FBS was added to each base well to act as an attractant to the cells in the upper insert. The chamber was then placed in a CO_2_ incubator at 37 ^◦^C. After 48 h of incubation, non-invasive cells on the upper surface of the chamber were removed by gently scraping with a cotton swab. The cells that successfully invaded the lower chamber were fixed using 4% formaldehyde and stained with 0.5% crystal violet. For quantification, the invaded cells were counted in five pre-determined fields using a DMi8 inverted microscope (Leica, Germany). The percentage of cell invasion inhibition was calculated by comparing it to the number of invaded cells in the untreated group. ImageJ software (NIH, USA) was used to quantify the number of invaded cells in each well.

### Colony formation

The long-term proliferative capacity of MDA-MB-231 and BT-20 cells was assessed using a colony formation assay, as described by our group^106,107^. Briefly, a 0.4% noble agar solution was prepared by mixing it with complete DMEM culture medium supplemented with 10% FBS. This mixture (1mL) was poured into each well of a 6-well plate to form a solid base layer. Once the agar solidified and dried, a top layer consisting of 0.3% agar mixed with 5,000 cells per well, with/without treatment, was added over the base layer. Cells were treated once at the time of plating with the indicated compounds or DTX at 2 µM. Colony formation was observed over a period of 3 weeks, during which images were captured from various areas of each well. The colonies were imaged using a Leica inverted microscope (Leica Microsystems, Germany). Results were expressed as the number of colonies per well and compared to the DMSO-treated control group.

### Western blot

Western blotting was performed to assess the expression levels of target proteins in MDA-MB-231 and BT-20 cells following treatment with 2 µM of **OH17**, **OH25** and DTX for 48 h. Proteins were extracted from treated and untreated cells, and then the concentrations were determined using a BCA protein assay kit (Thermo Fisher Scientific, USA). Equal amounts of total protein (30 µg) were resolved on 10% SDS-PAGE prepared from TGX™ FastCast™ Acrylamide Kit (BioRad, USA), followed by blotting on a PVDF transfer membrane, 0.45 μm (Thermo Scientific, USA). Membranes were blocked with 5% BSA (Thermo Scientific, USA) in Tris-buffered saline with 0.1% Tween-20 (TBST) for 1 h at room temperature and then incubated overnight at 4 °C with primary antibodies. The primary antibodies used in this investigation are listed in the supplementary file (Table S1). On the next day, membranes were washed with 1x TBST buffer and incubated with appropriate HRP-conjugated secondary antibodies for 2 h at room temperature. Then the membranes were incubated with Pierce™ ECL Western Blotting substrate (Thermo Scientific, USA), and visualized using iBright Imaging Systems (Thermo Scientific, USA). Densitometric analysis was performed using ImageJ software, and target protein levels were normalized to the corresponding housekeeping protein.

### Statistical analysis

All experimental data were analyzed using GraphPad Prism (Version 10, GraphPad Software, USA). Results are presented as mean ± standard error of the mean (SEM) from at least three biological independent experiments (*n* = 3). Differences between treated and untreated control (CTL) were determined using one-way analysis of variance (ANOVA) followed by Tukey’s post-hoc test. Dose-response curves were generated, and the half-maximal inhibitory concentration (IC₅₀) values were computed using a nonlinear regression test. The selectivity index (SI) was calculated using IC₅₀ values derived from cell viability assays, according to the formula: SI = IC₅₀ (normal cell line)/IC₅₀ (TNBC cell line). Results were considered statistically significant when *p-values* were < 0.05.

## Supplementary Information

Below is the link to the electronic supplementary material.

Supplementary Material 1

## Data Availability

Data is provided within the manuscript or supplementary information files.
