# Supplementary material for: Novel pyridine-based chalcone analogs and triple negative breast cancer: potential therapy & molecular pathways
Source: Sci Rep. 2026 May 30;16:24800. doi: 10.1038/s41598-026-51789-0 (PMC13458508; doi:10.1038/s41598-026-51789-0)
Supplement: Supplementary file 1 [file 41598_2026_51789_MOESM1_ESM.pdf]

# Supplementary File

Table S1. List of Western Blot primary antibodies used in our study.

| **Antibody** | **#Catalog number** | **Manufacturer** |
|---|---|---|
| Anti-ERK1/2 | 4695S | CST, USA |
| Anti-p-ERK1/2 | 4370S | CST, USA |
| Anti-EGFR | 4267S | CST, USA |
| Anti-p- EGFR | 3777S | CST, USA |
| Anti-Bax | 5023S | CST, USA |
| Anti-Bcl-xl | 2762S | CST, USA |
| Anti-Caspase-3 | 9662S | CST, USA |
| Anti-P53 | 2527S | CST, USA |
| Anti-c-Jun | AB32385 | Abcam, UK |
| Anti-GAPDH | 5174S | CST, USA |

Table S2. Summary of treatment conditions used for each assay.

| **Assay** | **Cell line used** | **Used compounds** | **Concentration used** | **Treatment duration** | **Fixed or cell-line-specific dose** |
|---|---|---|---|---|---|
| General Screening | MDA-MB-231 and BT-20 | 14 compounds (**OH13** to **OH26**) | 2.5 μM for all compounds | 48 hours | Fixed |
| Cell Viability Assay | MDA-MB-231, BT-20 and MCF-10A | **OH17** **OH25** DTX | Range of concentrations from (0-6μM) | 48 hours | Fixed |
| Morphological examination | MDA-MB-231, BT-20 and MCF-10A | **OH17** **OH25** DTX | 2 μM (For all compounds) | 48 hours | Fixed |
| Cell cycle | MDA-MB-231 and BT-20 | **OH17** **OH25** DTX | 2 μM (For all compounds) | 48 hours | Fixed |
| Apoptosis (Annexin V/PI) | MDA-MB-231 and BT-20 | **OH17** **OH25** DTX | 2 μM (For all compounds) | 48 hours | Fixed |
| Invasion Assay | MDA-MB-231 and BT-20 | **OH17** **OH25** DTX | 2 μM (For all compounds) | 48 hours | Fixed |
| Colony formation | MDA-MB-231 and BT-20 | **OH17** **OH25** DTX | 2 μM (For all compounds) | 48 hours | Fixed |

| | | | | | |
|---|---|---|---|---|---|
| Western blot | MDA-MB-231 and BT-20 | **OH17**<br>**OH25**<br>DTX | 2 μM<br>(For all compounds) | 48 hours | Fixed |

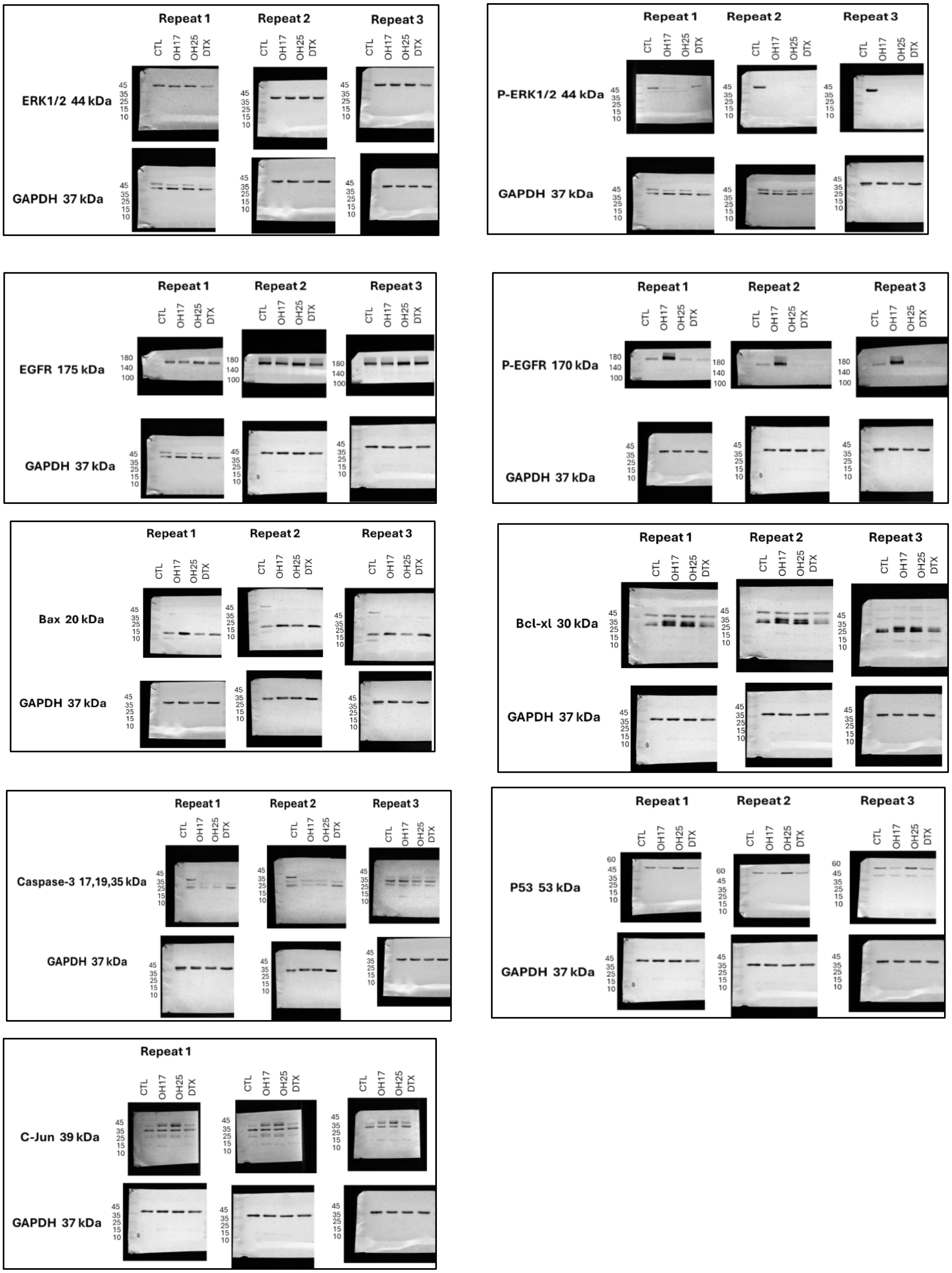


Figure 1. Shows the whole blot after cutting membrane at molecular weight 100 kDa. Original cropped membranes for MDA-MB-231 cells treated with OH17, OH25, and DTX at 2 µM.

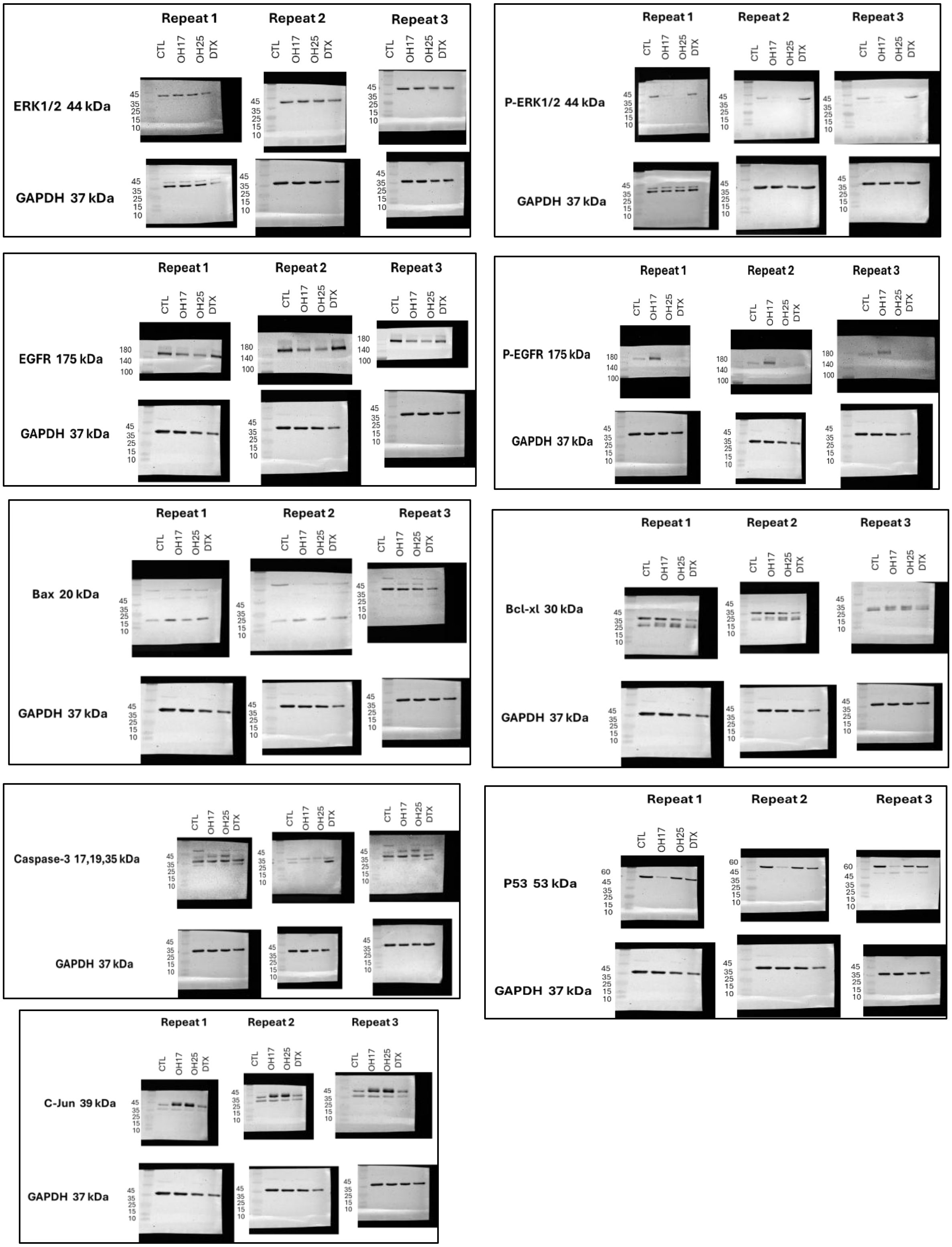


Figure 2. Shows the whole blot after cutting membrane at molecular weight 100 kDa. Original cropped membranes for BT-20 cells treated with OH17, OH25, and DTX at 2 µM.
